# Impact of Structured Rounding Tools on Time Allocation During Multidisciplinary Rounds: An Observational Study

**DOI:** 10.2196/humanfactors.6642

**Published:** 2016-12-09

**Authors:** Joanna Abraham, Thomas G Kannampallil, Vimla L Patel, Bela Patel, Khalid F Almoosa

**Affiliations:** ^1^ Department of Biomedical and Health Information Sciences University of Illinois at Chicago Chicago, IL United States; ^2^ Department of Family Medicine University of Illinois at Chicago Chicago, IL United States; ^3^ Center for Cognitive Studies in Medicine and Public Health New York Academy of Medicine New York, NY United States; ^4^ Division of Critical Care Medicine Department of Internal Medicine University of Texas Health Science Center Houston, TX United States; ^5^ Memorial Hermann Katy Hospital Katy, TX United States

**Keywords:** teaching rounds, communication, intensive care units

## Abstract

**Background:**

Recent research has shown evidence of disproportionate time allocation for patient communication during multidisciplinary rounds (MDRs). Studies have shown that patients discussed later during rounds receive lesser time.

**Objective:**

The aim of our study was to investigate whether disproportionate time allocation effects persist with the use of structured rounding tools.

**Methods:**

Using audio recordings of rounds (N=82 patients), we compared time allocation and communication breakdowns between a problem-based Subjective, Objective, Assessment, and Plan (SOAP) and a system-based Handoff Intervention Tool (HAND-IT) rounding tools.

**Results:**

We found no significant linear dependence of the order of patient presentation on the time spent or on communication breakdowns for both structured tools. However, for the problem-based tool, there was a significant linear relationship between the time spent on discussing a patient and the number of communication breakdowns (*P*<.05)––with an average of 1.04 additional breakdowns with every 120 seconds in discussion.

**Conclusions:**

The use of structured rounding tools potentially mitigates disproportionate time allocation and communication breakdowns during rounds, with the more structured HAND-IT, almost completely eliminating such effects. These results have potential implications for planning, prioritization, and training for time management during MDRs.

## Introduction

Multidisciplinary rounds (MDRs) serve as a common venue for formulating shared patient care goals and plans of care by care providers from different clinical specialties [1,2]. Due to its multidisciplinary and collaborative format, MDRs support a patient-centered model of care [3,4]. Studies on MDRs have demonstrated positive clinical outcomes through improvements on patient care quality and safety [5], minimization of hospital length of stay (LOS) [6], and reduction in patient mortality rates [7]. In addition to their prominent role in care coordination [8,9], MDRs provide a forum for discussing diagnoses and treatment trajectories and practicing communication and professional skills. In critical care units, MDRs are often the only forum for the transfer of patient care responsibilities [10].

Notwithstanding these patient care benefits, researchers have pointed out several divergent perspectives regarding MDRs [11-14]. Much of the prior research has reported on the frequency and duration of rounds [15-17]. For example, Plantinga et al [17] reported that patients who had more frequent sit-down MDRs had better health outcomes, often achieving their clinical performance targets. Similar studies on MDRs in trauma settings have shown an increase in the efficiency of patient flow, reduction in length of stay, and unnecessary revisits [16]. Although there is evidence that planned and structured MDRs, especially in critical care settings, can improve patient and clinician outcomes [15], a major concern regarding MDRs is the time taken away from patient care activities [1].

One of the underexplored areas of research on MDRs is related to the time allocation and distribution during patient discussions. Recent research has illustrated that verbal discussions during rounds were vulnerable to unequal time allocation––a phenomenon that has been described as a “portfolio problem” or “end of round time compression” [18-20]. For example, Cohen et al [18] examined video-recordings of 23 end-of-week handoff sessions in a 21-bed intensive care unit (ICU) and found that patients discussed earlier received about 50% more time than the patients discussed later in the same session, regardless of their severity or complexity of illness. Similarly, Kannampallil et al, [20] reported an average decrease of 54 seconds for every additional patient discussion during morning rounds in a Cardiothoracic ICU. As a part of a larger clinical trial, Sung et al [21] analyzed 759 patient discussions from 2 clinical teams and found similar decrease in the time spent for patients discussed later during the rounds, after adjusting for illness severity.

The presence of such a disproportionate allocation of time can lead to potential decision-making and communication failures, with a consequent detrimental impact on care coordination and safety outcomes [22,23]. The causal underpinnings of such a temporal phenomenon have been debated, but require further exploration [19,24]. To support effective and efficient decision-making and communication during MDRs, hospitals have relied on a wide range of rounding tools [1,25,26]. These include patient-centric tools, which help clinicians to gather information on the clinical condition of a patient; process-oriented tools, which help clinicians to organize information to support verbal communication during rounds; and decision-support tools, which help clinicians to make decisions related to clinical diagnosis and treatment.

In this exploratory study, we evaluate the effect of 2 structured rounding tools on time allocation for patient case presentation and communication during daily rounds. As a secondary research question, we also examine whether the distribution of time allocation has an impact on the effectiveness of round communication.

## Methods

The data used for this study were collected as part of a larger study that compared the communication practices in a medical ICU (MICU) [27,28].

### Study Setting

This study was conducted in a 16-bed MICU at a tertiary medical center with approximately 55,000 emergency department visits per year. This MICU follows a “closed” model of care, where patient care decisions are internally managed by the MICU multidisciplinary team comprising an attending physician (ie, intensivist), a fellow, residents and interns, critical care nurses, a pharmacist, a respiratory therapist, and a nutritionist. The MICU residents’ and interns’ shifts lasted for approximately 24 hours, with additional 4 hours for participating in care transition activities during rounds (from ~8:00 am, day 1 to ~12:00 pm, day 2).

The unit has an average of 1200 patient admissions per year (Case Mix Index=4.72; average patient LOS=3.8 days; average number of vent days=3.1; and top 2 diagnosis-related group codes were sepsis and respiratory failure).

### Rounding Process

The formal morning MDRs were led by an attending physician, and focused on transferring information, responsibility, and control from the outgoing team (postcall resident and intern) to the incoming team (on-call resident and intern). At this setting, there were no formal protocols and practices on the selection of the order of patient case presentations during rounds.

### Rounding Tools

Two paper-based rounding tools were used: a patient problem-oriented, Subjective, Objective, Assessment, and Plan (SOAP) note, and locally developed, body systems-oriented, Handoff Intervention Tool (HAND-IT) [27]. The rounding tools (SOAP or HAND-IT) were used for gathering patient care information in preparation for rounds, and for supporting presentation and communication during MDRs.

**Figure 1:**
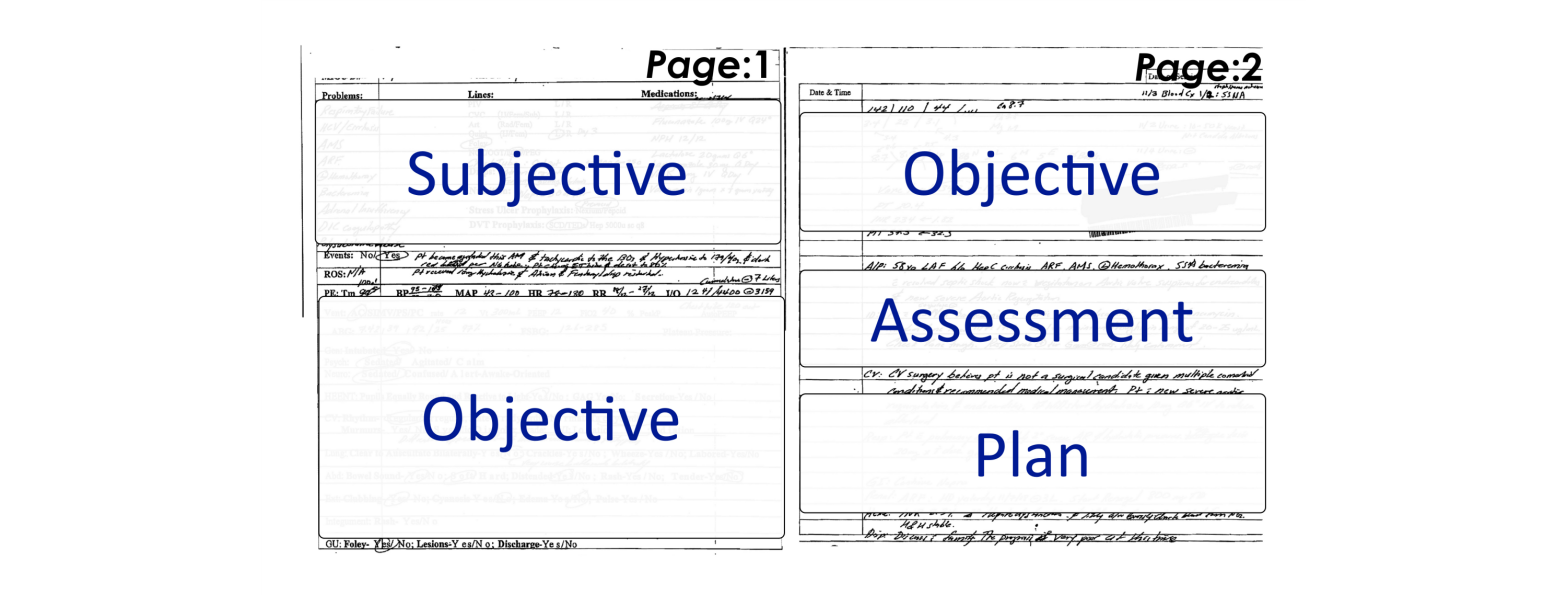
Subjective, Objective, Assessment, and Plan (SOAP)-based tool that was used for the rounds.

SOAP is based on the problem-oriented medical record format [29]. The SOAP tool aids physicians to focus on the primary complaints of the patient, and other care-related information categorized under 4 headers (Figure 1). Subjective information regarding the patient includes patient’s chief complaint and history of patient illness including past and pertinent medical, family, and social history. The objective component comprises information gathered through observations of patient actions and behaviors including physical exam, and results from laboratory and radiology tests pertinent to the current episode of care. The assessment comprises the clinical impression regarding the patient case summarized for the newest or most acute problem including a statement of patient problem, differential diagnosis, and reasoning regarding the problem. Assessment is often based on the subjective and objective data, and indicates progression of change or no change in patient condition. Finally, plan comprises 4 separate information categories such as diagnostic testing, treatment plan, patient education, and planned follow-up, listed for all patient problems [30].

**Figure 2:**
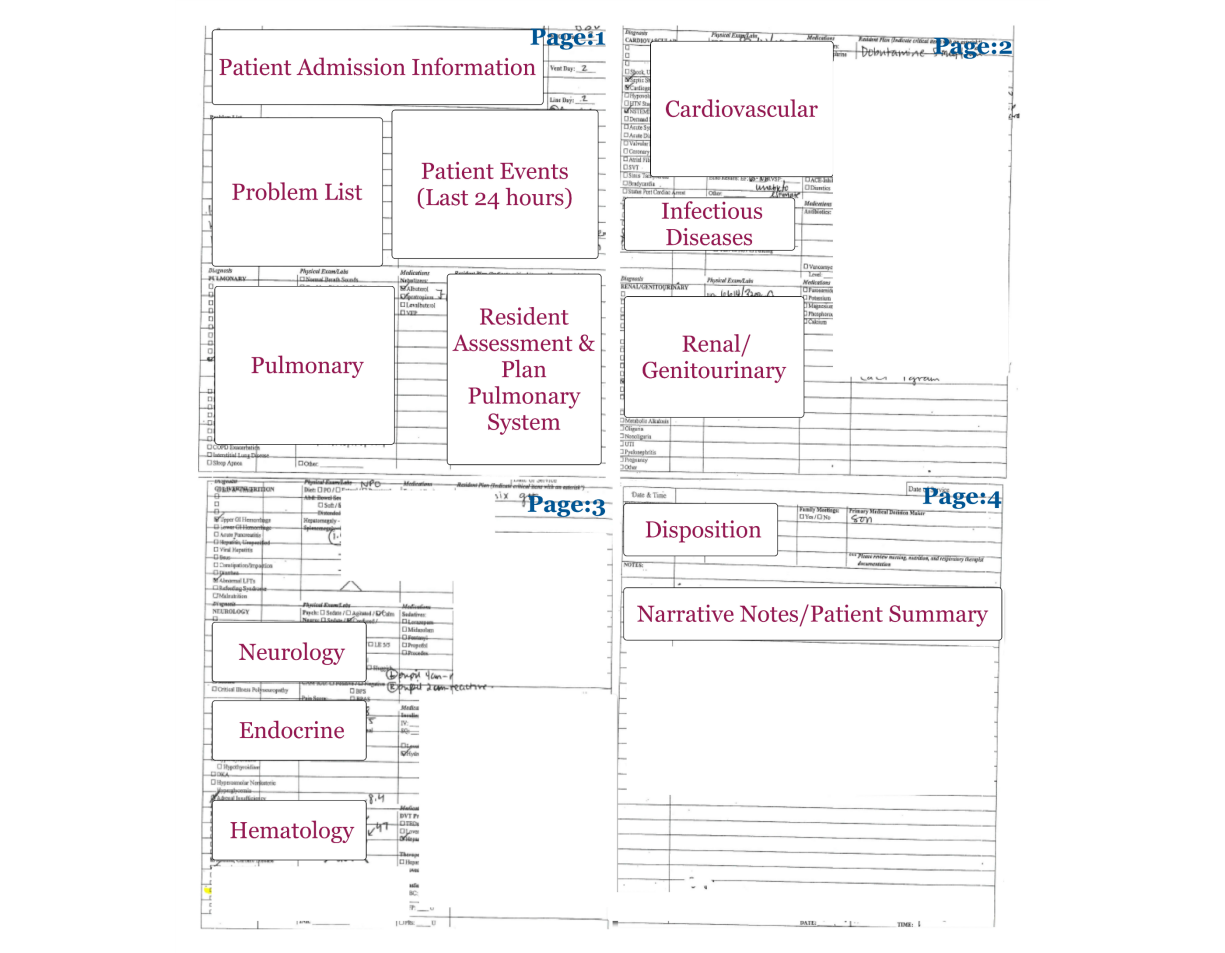
Body systems-oriented Handoff Intervention Tool (HAND-IT) with the various body system elements highlighted.

HAND-IT was developed based on a previous evaluation study that showed that structuring information in a checklist-based, body-systems format improves filtering, retrieval, and documentation of information in preparation for rounds [28]. The information on HAND-IT is organized by body systems including pulmonary, neurology, endocrine, hematology, cardiovascular, infectious disease, and renal and genitourinary organ systems. The information within each body system is organized in a medical knowledge hierarchical format [31]. Such an organization helps physicians in developing a bottom-up understanding of a patient case: in other words, this format supports inductive reasoning helping physicians in translating clinical data to clinical hypothesis, leading to effective treatment or management decisions [31,32]. HAND-IT also follows the Society of Critical Care Medicine’s guidelines including identification of delirium, sedation practices, prophylaxis, and feeding information [33] (Figure 2).

### Participants

There were 16 participants during the 2-month study period, divided into 2 independent teams. Each team was in the MICU for a 1-month period and consisted of 8 core participants for the entire month (1 attending physician, 1 fellow, 3 residents [PGY2/3], and 3 interns [PGY1]). In addition to this, there were 6 critical care nurses, 1 pharmacist, 1 respiratory therapist, and 3 medical students who participated in the rounds each month. The institutional review board of the University and Hospital approved this study and written consents were obtained from all participants.

### Study Design and Data Collection

Morning rounds on 8 randomly selected days over the course of 2 months with 2 independent MICU care teams were audio recorded. The recordings consisted of round discussion of 82 patient cases (n_SOAP_=41, n_HAND-IT_=41). Follow-up informal interviews with physicians confirmed that the order of patient presentation and discussion varied depending on the attending physician’s priority and patient acuity.

During the first month of data collection, team 1 trained with SOAP for 4 days, followed by 2 days of testing; then trained with HAND-IT for 4 days, followed by 2 days of testing. During the second month, a new team followed the same process of training and testing with the reverse order of tool usage (ie, HAND-IT followed by SOAP). This was done to counterbalance the effects of tool use. The training period involved introductory training on the structure and various content fields of each tool. During the training period, residents used their assigned tool during rounding to gain familiarity.

**Figure 3:**
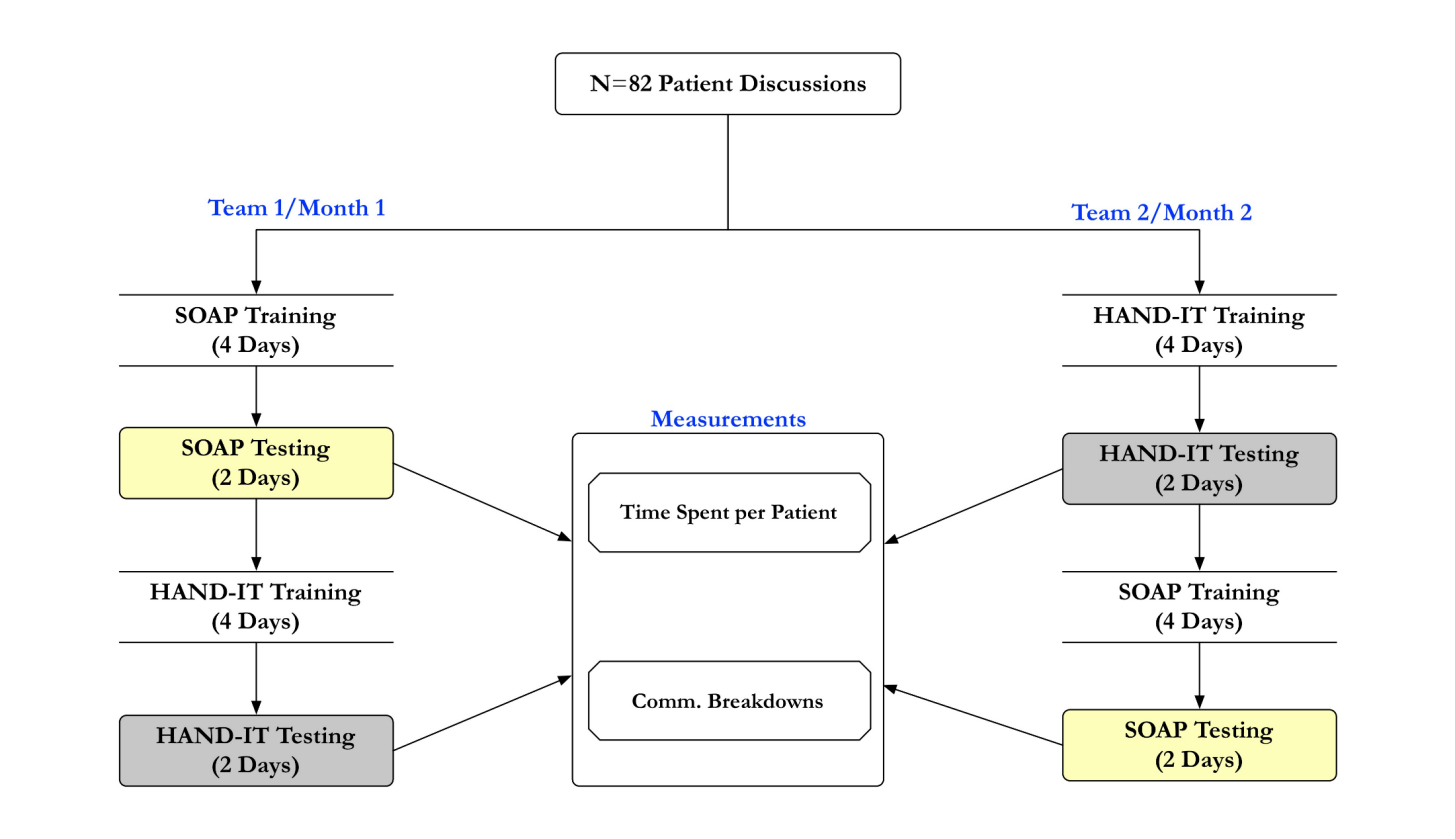
The study design showing the organization of training and testing using both tools is shown. The measurements (time spent and communication breakdowns, shown in the center) were compared with the order of patient case presentation. SOAP: Subjective, Objective, Assessment, and Plan; HAND-IT: Handoff Intervention Tool.

The testing period involved collection of verbal communication data through audio recording of the rounds. The total audio recorded time was approximately 40 hours. In addition, a researcher (the first author, JA) observed these sessions, made field notes, and conducted informal interviews after the rounds. An illustrative representation of the study design is shown in Figure 3.

Given the exploratory nature of this study, and limited previous research results, our purpose was to compare our results with the results reported in other published research articles [18]. There was no control condition (ie, a “no tool” condition), and the comparisons were made only between the 2 considered rounding tools.

### Data Coding

Audio-recorded verbal communication during rounds was used to compute the length of time spent presenting each patient. The verbal transcripts were used to evaluate the quality of communication during rounds.

#### Round Communication Duration

Two researchers (the first author, JA, and a research assistant) listened to audio recordings to note the time spent on discussing each patient. The start time of each handoff was identified as the moment when the resident or intern started a patient presentation. The end time of each handoff was identified as the moment when the attending physician signed-off on his or her progress note for a patient case. This denoted the end of patient discussion. The audio recordings were also marked-up for interruptions, and other distractions unrelated to the patient case being presented. The total time was calculated by combining the duration of patient presentation and discussion, and excluding the time periods of interruptions, similar to the time coding performed by Cardarelli and colleagues [34].

There was a significantly high inter-rater agreement between the 2 coders (Cohen *κ*=.975). The discrepancies in the time identification were resolved through discussion and relistening to the audio recordings.

#### Round Communication Breakdowns

Breakdowns were defined as any failure in information flow and transfer from the outgoing postcall team to the on-call team (ie, receiving team consisting of the attending physician, fellow, resident, intern). The breakdowns in communication were evaluated using a validated communication framework [8,27] and classified into one of the following 3 categories: missing or incomplete information, incorrect or conflicting information, and irrelevant or ambiguous information.

Two authors (JA, TK) coded the breakdowns in communication with a high degree of inter-rater agreement (Cohen *κ*=0.96). Any disagreement in the coding of breakdowns was resolved through discussion. Description of each of these types of breakdowns is shown in Table 1. Although we categorized breakdowns into 3 categories for coding purposes, we did not perform separate analyses for each type of breakdowns.

**Table 1:** Different types of communication breakdowns that were coded for each of the transcripts.

Type of communication breakdowns	Description
Incomplete information	Lack of complete patient information provided by the postcall team to the oncall team during rounds
Inaccurate and conflicting information	Erroneous patient information provided by the postcall team to the on-call team during rounds
Irrelevant information	Inappropriate care plan provided by the postcall team to the oncall team during rounds (that does not follow the clinical reasoning logic nor suitable for the patient at that moment in time)

### Statistical Analysis

To determine whether there was a significant relationship between the order of presentation of patient cases and the time spent on the discussion for each of the tools, we computed the Kendall τ rank order coefficient for each session. Kendall τ rank order coefficient is a nonparametric test statistic that is used to determine the measure of association between 2 variables. The test statistic provides a measure of the rank correlation between the ordering of data ranked by each of the variables. As the predictor variable is ordinal, Kendall τ provides an appropriate test regarding the hypothesized relation with values varying between −1.0 and +1.0. A negative Kendall τ between the order of presentation and time spent shows lesser time for patients presented later, zero correlation shows that relatively equal time was spent across all patients, and a positive correlation shows more time spent for patients presented later. Given that the data were collected across 8 sessions (4 sessions per tool), similar to Cohen et al [18], we computed the Kendall τ per session and averaged across all sessions per tool.

Similar rank order coefficients were also computed for evaluating whether the order of presentation had any effect on communication breakdowns for each of the tools. Linear regression analysis was also used to investigate the relationship between the time spent on patient discussion and communication breakdowns. A significance level of *P*<.05 was used.

## Results

There were no differences in the number of patients discussed per day between the 2 rounding tools (*t*=0, *P*>.05; Mean_SOAP_=10.3, SD=3.3; Mean_HAND-IT_=10.3, SD=2.2). In addition, there were no differences in the time spent on discussion of each patient between the 2 rounding tools (*t*=0.56, *P*>.05; Mean_SOAP_=770.9, SD=55.6; Mean_HAND-IT_=753.4, SD=110.3).

In terms of the time spent per patient with respect to the order of presentation, the mean (SD) Kendall τ correlations were marginally negative for SOAP (−0.11 [0.38]), and HAND-IT (−0.01 [0.30]). In terms of the communication breakdowns with respect to the order of presentation, the mean (SD) Kendall τ correlations were negative for SOAP (−0.25 [0.41]), and marginally positive for HAND-IT (0.05 [0.17]). In other words, the time spent on discussing a patient or the number of breakdowns did not change significantly over the course of a session for either rounding tool, potentially showing no disproportionate time allocation or communication breakdown effects.

**Figure 4:**
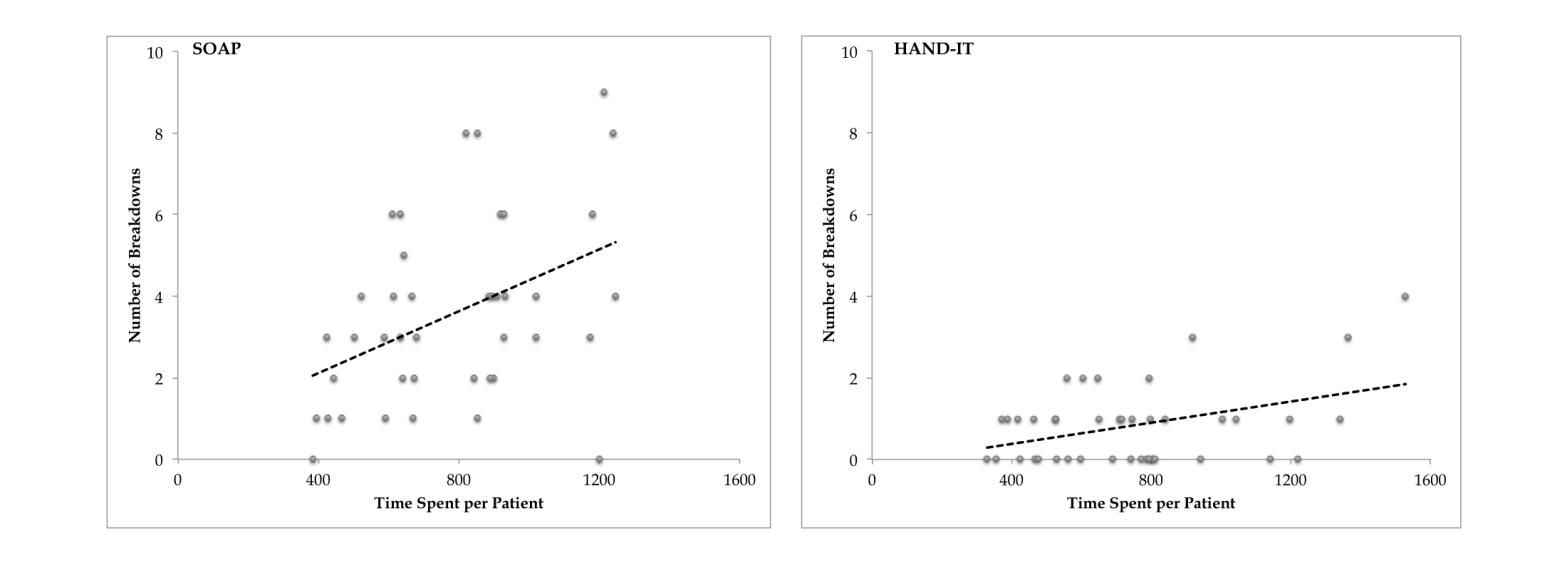
The number of breakdowns as a function of the time spent per patient for Subjective, Objective, Assessment, and Plan (SOAP) and Handoff Intervention Tool (HAND-IT) tools. For SOAP, the number of breakdowns increases (n=41 patient discussions)—the trend line for the estimated linear regression is b=.0038t+.59 (*P*<.05, 95% CI of t: 0.00118, 0.0064). For HAND-IT, the increase is marginal––the trend line for the linear regression is b=.0013t−.138 (*P*<.05, 95% CI of t: (0.00031, 0.0022). Both estimates were statistically significant at *P*<.05.

However, based on regression analysis, there was a significant linear dependence between time spent discussing patients and breakdowns (*P*<.05): for SOAP, there was an average increase of 1.04 breakdowns with every additional 120 seconds spent on discussing a patient. For HAND-IT, the increase in breakdowns was about 0.018 for a similar 120 seconds additional time spent on discussing a patient. In other words, the increased length of conversation per patient is more likely to lead to communication breakdowns in SOAP than in HAND-IT. The summary of the linear dependence between communication breakdowns and time spent on discussing the patients is shown in Figure 4.

## Discussion

### Principal Findings

Our results suggest that structured tools are likely to mitigate the effect of disproportionate time allocation during rounds. Although correlations of the order of presentation in relation to both time spent and breakdowns in communication were marginal for both HAND-IT and SOAP, the relative effect was lesser for HAND-IT: with almost no correlation; Kendall τ being .01 and .05 for time spent and breakdowns in communication, respectively. We also found that additional time spent in discussing a patient during MDRs may lead to more breakdowns in communication in SOAP than that in HAND-IT.

Although further research is required to ascertain how structured tools mitigate the disproportional time allocation across patients, we acknowledge that there would be instances where structured tools may not be strictly followed due to patient-, clinician-, and environmental-related factors in critical care settings, in which cases, disproportionality in time allocation may be preferred (eg, differences in patient complexity and acuity, number of days the patients has been in the unit, and recent changes in the patients’ condition).

We discuss 3 implications of our results within the context of the MDR process: supporting communication, planning for distribution of time, and prioritization of patient order. Research on rounds has focused primarily on developing tools for supporting information presentation by outgoing clinicians using an information transmission perspective [35], with limited functionalities to foster the tasks of information gathering and organization by incoming clinicians. Structured tools such as HAND-IT can serve as cognitive support for promoting effective communication, as it allows the incoming clinician to know what to expect during the presentation and to quickly identify any discrepancies or gaps in the ongoing communication and instantaneously repair them. In addition, our informal discussions with residents provided evidence that although HAND-IT required more effort and time to gather and document information, it reduced the time spent and additional effort during rounds to address the information gaps. .

Research in psychology and cognitive sciences has shown human limitations regarding planning for tasks––both in terms of biases in time allocation, and overconfidence in the precision of outcomes [36]. In other words, human planning for time is predicated on optimistic expectations of timely task completion, even with prior evidence to the contrary. During MDRs, uncertainties of time requirements are amplified by factors such as patient uncertainty, unexpected complications, varying clinician task load, multiple consult service coordination of care decisions, and possible new admissions (eg, transfers from emergency room or floor units). In addition to these contextual factors, there are organizational aspects that put a significant constraint on time availability for MDRs. For example, Accreditation Council for Graduate Medical Education (ACGME) guidelines on resident hours restrict maximum duty limit to 28 hours––24+4 hours for transitional and education activities––requiring rounds to be completed by a certain time. Such requirements add to the planning challenges. Structured tools can potentially help in streamlining conversations, smoothing the time spent across multiple patients in a session thereby helping in time planning and removing the element of “subjectivity” that is often attributed to personal physician preferences, style, and priorities [9].

Another closely related aspect of rounding is prioritization. Physicians often select and prioritize patients for discussion during MDRs. These selections are based on patient criticality (eg, the sickest patient first), time of admission (ie, LOS in the unit), bed order, or costeffectiveness ratio [37]. For example, Cohen et al [18] suggested that the sickest, newest, or patient’s requiring further discussions should be seen first during rounds. However, there are other external constraints that play into the decisions regarding the priority order of patient presentation that can accelerate the disposition of patients in a unit. Tools supporting such global strategies and assisting in patient prioritization have been described to improve efficiency in critical care settings [38]. In an another study, Iapichino et al [39], suggested stratification of patients in intensive care settings should be based on their illness severity at patient admission to achieve cost effectiveness in the care delivery process.

### Limitations

We acknowledge that this exploratory study has several limitations.

First, the study was conducted at a single academic MICU setting using a nonrandomized design with only 2 clinical teams. However, we evaluated a large number of handoffs (N=82 patients) providing validity for our preliminary results.

Second, we did not control for any patient-related, unit-related, or other external variables in our analysis. Our assumption was that, given the unpredictability of patient arrivals or discharges and similar resource availability for all patients, the order of patient discussion was effectively randomized, making any of the patient, unit, or external variables unrelated to the discussion order (a similar claim was made by Cohen et al [18] regarding randomization and discussion order).

Third, the increased number of breakdowns for longer communications may have been an effect of length-biased sampling: the greater the length of the conversation, the greater the likelihood of communication breakdowns.

Fourth, in this study, we did not have a true “control” condition; that is, a condition where we showed the existence of disproportionate time allocation during rounds. Instead, drawing on a prior study—by one of the coauthors [20] —and on recently reported research literature that showed the evidence for disproportionate time allocation, we evaluated whether structured tools had any effect on moderating the effects of disproportionate time allocation.

Finally, although our exploratory findings demonstrate the moderating effects of structured rounding tools on time allocation, we would like to acknowledge that at times, disproportionate time allocation maybe unavoidable. Such situations arise due to complexity of patient cases, LOS of patient, prior knowledge of the patient, limited changes in therapeutic regimen, or other time constraints.

### Conclusions

Time constraints impose challenges to critical care practice, often adding additional cognitive load on the physician’s already complex work activities. One of the unintended effects of time constraints is their disproportionate time allocation to similar tasks. Although there is no evidence on whether disproportionate time allocation can have any detrimental outcomes, it increases the possibility for errors and inefficient patient care delivery and management. We found preliminary evidence that structured rounding tools may mitigate such disproportionate time allocation effects during MDRs. In addition, increased structure within the tools can also mitigate the communication breakdowns during MDR discussions. Although our results provide preliminary evidence of the time allocation and quality of communication using structured tools, further research is required to establish the causal underpinnings of time allocations during rounds.

## Abbreviations

HAND-IT: Handoff Intervention Tool
ICU: intensive care unit
LOS: length of stay
MDRs: multidisciplinary rounds
MICU: Medical ICU
SOAP: Subjective, Objective, Assessment, and Plan
